# Lipid-Induced Oxidative Modifications Decrease the Bioactivities of Collagen Hydrolysates from Fish Skin: The Underlying Mechanism Based on the Proteomic Strategy

**DOI:** 10.3390/foods13040583

**Published:** 2024-02-14

**Authors:** Fengjie Gou, Song Gao, Bo Li

**Affiliations:** Beijing Laboratory for Food Quality and Safety, College of Food Science and Nutritional Engineering, China Agricultural University, Beijing 100083, China; gxb992022@163.com (F.G.); gaosong36@cau.edu.cn (S.G.)

**Keywords:** collagen peptides, oxidation, amino acid modification, bioactivity, proteomic

## Abstract

Collagen peptides exhibit various bioactivities, including antioxidation and ACE inhibition. However, the bioactivities of collagen peptides decrease gradually due to oxidation deterioration during storage, and this degradation of bioactive peptides is rarely studied. In this study, the oxidative levels and the bioactivities of collagen peptides were investigated during an oxidative-induced storage accelerated by lipids. The results suggested that the oxidation of collagen peptides was divided into three stages. At the early stage, the carbonyl content of collagen peptides increased rapidly (from 2.32 to 3.72 μmol/g peptide), showing a close correlation with their bioactivities (for antioxidation, r = −0.947; for ACE inhibition, r = −0.911). The oxidation level in the middle stage continued but was stable, and the bioactivities decreased. At the later stage, the Schiff base and dityrosine content increased significantly and showed a strong correlation with the bioactivities (antioxidation, r = −0.820, −0.801; ACE inhibition, r = −0.779, −0.865). The amino acid and proteomic analyses showed that Met, Lys, and Arg were susceptible to oxidation and revealed their oxidative modification types. This study provided an insight into the dynamic oxidative modifications of collagen peptides, which were shown to correlate well with the change in bioactivities.

## 1. Introduction

Collagen peptides (CPs) have great potential for being developed into functional food ingredients, with the advantages of low molecular weight, high absorption, and bioavailability [1]. In addition, it has been found that peptides derived from collagen hydrolysates exhibit a variety of bioactivities in vitro or in vivo, including antioxidation [2], antihypertension [3], anti-inflammation [4], antiplatelet aggregation [5], and anti-osteoporosis [6]. However, a variety of food-related molecules, such as aldehydes and hydroxyl radicals, can react or interact with peptides, resulting in a decrease in their bioavailability or bioactivity [7], which may limit the applications of bioactive peptides in the food industry.

Several studies on the bioactive stability of protein hydrolysates during storage have been reported [8,9]. After 28 days of storage, the contents of histidine (His), tyrosine (Tyr), tryptophan (Trp), and lysine (Lys) in egg-white peptides reduced significantly, leading to a decrease in the antioxidant activity [10]. Degradation of specific amino acids (Trp, His, and Tyr) in casein hydrolysates and casein polymerization were observed after 28 days of storage under continuous light exposure, which could be attributed to the decrease in ACE-inhibitory activity [11]. The occurrence of these decreases in activity is linked to oxidative damage. Such oxidative behaviors are associated with a variety of factors such as reactive oxygen species (ROS) [12], the Maillard reaction [13], and lipid oxidation [14]. Although a previous study has shown that lipid oxidation enhances protein oxidation during storage [15], the extent to which the underlying mechanism of this oxidation influences the biological activity of peptides remains unclear.

Fish skin, which is rich in collagen, is an important source for the preparation of CPs [16]. However, the unsaturated fatty acids in fish skin undergo oxidation and degradation during collagen extraction and enzymatic hydrolysis [14]. These lipid alterations not only result in the generation of volatile aldehydic compounds and fishy odor [5], but also exacerbates the oxidative damage of proteins [17]. Such lipid-induced oxidation could play a major role in the diminished activity of bioactive peptides during processing and storage. Furthermore, oxidation of the side chains in some amino acids (Lys and Arg) leads to the formation of carbonyls, which might affect the biological activity of the peptides [7].

Therefore, this study aimed to understand how lipid-induced oxidation can induce chemical modifications in collagen peptides that could affect their bioactivities. Specifically, the study explored the oxidation behavior of collagen peptides in the co-induced oxidation by fish oil and H_2_O_2_ based on an accelerated storage trail (37 °C). Carbonyl compounds and fluorophores were used as markers for the oxidation of peptides, and the LC-MS/MS method was employed to characterize the oxidation sites of collagen peptides. This study hypothesized that the amino acid modification products induced by the oxidation of unsaturated lipids at different storage stages were responsible for decreases in antioxidant activity and ACE-inhibitory activity.

## 2. Materials and Methods

### 2.1. Materials and Chemicals

Gelatin from tilapia skin was purchased from Yiweilong Biotechnology Co., Ltd. (Xiamen, China). The protease Protamex was obtained from Novozymes Biotechnology Co., Ltd. (Beijing, China). Fish-oil soft capsules (0.12 g DHA + 0.18 g EPA/1.0 g fish oil) were purchased from Swisse Wellness PTY LTD (Melbourne, Australia). Dialysis bag MD34 (MW: 200) was obtained from Mai Shang Scientific Instrument Company (Shanghai, China). Other chemical reagents used in this study were analytical grade or better.

### 2.2. Preparation of CPs and Molecular Weight (MW) Distribution

Collagen (10%, *w*/*v*) was hydrolyzed by Protamex (2%, *w*/*w*) at 50 °C for 6.0 h (pH 7.0), and then the collagen hydrolysates were dialyzed to remove free amino acids and salts. The collagen hydrolysate was placed in an MD34 dialysis bag (MW: 200), and then this bag was immersed in deionized water at 4 °C for 24 h. The CPs were prepared for further analysis after being freeze-dried.

The MW distribution of the CPs was evaluated as described by Zhang et al. [18]. An isocratic elution was performed using a mobile phase (acetonitrile and water containing 0.1% (*v*/*v*) trifluoroacetic acid (TFA), 45:55, *v*/*v*). After being filtered through a 0.22-μm microfiltration membrane, the samples of collagen hydrolysates or CPs (2 mg/mL, 10 μL) were injected onto a TOSOH TSK gel G2000 SWXL column (7.8 × 300 mm, Tokyo, Japan), and then they were eluted with TFA-acetonitrile buffer at a 0.5 mL/min flow rate. A UV detector (220 nm) was used for sample analysis and quantification. Standards from Sigma, such as aprotinin (6512 Da), bacitracin (1423 Da), Tyr-Pro-Trp-Tyr (YPWY, 627 Da), Pro-Leu-Asp (PLD, 343 Da), and Gly-Pro (GP,172 Da), were used to obtain a MW calibration curve (y = −0.2633x + 7.9441; y: log MW; x: time, R^2^ = 0.99) to analyze the MW distribution.

### 2.3. Induction Methods of Oxidation

Two portions of CPs (400 mg) were dissolved in 10 mL PBS buffer (20 mM, pH 7.4) or PBS–Oil buffer (20 mM, pH 7.4, containing 1% fish oil, and two drops of Tween-20), respectively. The group with the presence of CPs and fish oil was named “CPs in the oxidation system” (CPOS), and the group with the absence of fish oil was the control (Control). The 10 mL PBS–Oil buffer was used as the blank (Blank). Sodium azide (0.05%, *w*/*v*) was added to all groups to inhibit microorganisms in the system.

Oxidation reactions in CPOS were conducted using a modified method from Xu et al. [19]. CPOS and Blank samples were homogenized with a high-speed disperser at 9200 rpm for 2 min and then incubated with 1 mM H_2_O_2_ (final concentration in emulsion) in a water bath at 37 °C for 24 h. Control samples were also incubated in a water bath at 37 °C for 24 h. All groups were shaken constantly, and equal amounts from each were taken at fixed times (0, 1, 2, 4, 8, 16, and 24 h).

The oxidized peptides were extracted from CPOS by 3 times the volume of n-hexane twice (at 4 °C for 24 h) and centrifuged (5000× *g*, 10 min); these were named Sample and were collected from the lower layer of solution.

After the oxidation reaction, CPOS, Blank, and Control aliquots from different times (0, 1, 2, 4, 8, 16, and 24 h) were directly measured for lipid oxidation levels, while Control and Sample aliquots were freeze-dried and redissolved for further analysis.

### 2.4. Measurement of Oxidation Levels of Lipid and Peptides

#### 2.4.1. Lipid Oxidation

The oxidation level in the CPOS, Blank, and Control groups was measured by the method of Zhou et al. [20], which was characterized by thiobarbituric acid-reactive substances (TBARS). A volume of 2.0 mL of reaction solution A (50 mM PBS containing 0.1% propyl gallate and 0.1% EDTA) was used to dissolve the sample (1.0 mL). This solution was then combined with 2.0 mL of reaction solution B (15% TCA and 0.375% TBA dissolved in 0.25 M HCl). After 20 min of heating in boiling water, the mixture was allowed to cool to room temperature. Following a 10 min centrifugation at 2000× *g*, the supernatant’s absorbance was determined at 532 nm. Lipid oxidation was assessed using the malondialdehyde standard curve (y = 0.4878x + 0.0075, R^2^ = 0.99).

#### 2.4.2. Total Sulfhydryl Content in CPs

The Ellman method was employed to estimate the sulfhydryl content [21]. Briefly, 1.0 mL of 0.2 M Tris-HCl buffer (containing 8.0 M urea, 1% SDS, and 3.0 mM EDTA, pH 8.0) was combined with 0.1 mL Control or Sample respectively, then vortexed for 30 s and mixed on a shaky platform for one hour. The mixture was then reacted with 0.1 mL 5,5′-dithiobis- (2-nitrobenzoic acid) (DTNB, 10 mM) in a darkroom for one hour. Following that, each sample was centrifuged at room temperature at 13,600× *g* for 15 min. At 412 nm, the absorbance of the supernatant was determined while sample blanks were recorded concurrently. A molar extinction value of 13,600 M^−1^·cm^−1^ was employed to determine the total sulfhydryl content.

#### 2.4.3. Carbonyl Content in CPs

The carbonyl contents in the Control and Sample groups were estimated by a modified method [22]. The solution of 2,4-dinitrophenylhydrazine (DNPH, 0.1% *w*/*v*, 0.02 mL) in 2 M HCl was combined with 0.1 mL of the samples, and the mixtures were left to derivatize for one hour in a dark room. TCA solution (20% *w*/*v*, 0.2 mL) was used to precipitate the peptides from Control or Sample, and the mixture was centrifuged at 5000× *g* for 5 min. The sediment was cleaned three times with 0.2 mL solution of ethyl acetate/ethanol (1:1, *v*/*v*). Ultimately, 6.0 M guanidine hydrochloride (0.6 mL, in 20 mM PBS) was used to re-dissolve each sample. The mixture’s absorbance was evaluated at 370 nm. A molar extinction coefficient of 22,000 M^−1^ cm^−1^ was used to calculate the carbonyl content. The results were presented in terms of μmol carbonyl/g peptide.

#### 2.4.4. Schiff Base Content in CPs

The content of Schiff bases was measured based on a previous method [23]. Peptides from Control or Sample were dissolved in 20 mM PBS (1 mg/mL). The excitation wavelength was set at 350 nm. Emission spectra were recorded in the wavelength range of 400 to 500 nm with the slit widths of excitation and emission at 10 nm. Data were obtained at 500 nm/min. The fluorescence intensity emitted at 460 nm was used to express the results.

#### 2.4.5. Dityrosine in CPs

The dityrosine content was measured according to the protocol of Davies et al. [24]. Peptides (10 mg) from Control or Sample were dissolved in 20 mM PBS (10 mL, pH 7.0). The fluorescence intensity of dityrosine was evaluated by a fluorescence spectrophotometer (Model F-4500, Hitachi, Tokyo, Japan). Excitation and emission wavelengths were 325 nm and 420 nm, respectively (bandwidth = 5 nm). PBS (20 Mm, pH 7.0) was used as a reagent blank. The difference in absorbance between the samples and the reagent blank was used to indicate the dityrosine content.

### 2.5. Determination of Biological Activity

#### 2.5.1. Antioxidant Activity

The ABTS^•+^ radical-scavenging activity was measured by a modified method of You et al. [25]. Firstly, potassium persulfate solution (2.45 mM) was mixed with ABTS^•+^ solution (7.0 mM) (1:1) to make the ABTS^•+^ storage solution. Then the storage solution was kept in a dark environment for 16 h. Subsequently, the working solution was obtained by diluting the ABTS^•+^ storage solution with 0.01 M PBS (pH 7.4) to an absorbance of 0.70 ± 0.02 at 734 nm. Finally, 50 µL of peptides from Control or Sample (1.5 mg/mL, pH 7.0) was mixed with 150 µL of the ABTS^•+^ working solution. The mixtures were incubated at 25 °C for 30 min in a dark room. The deionized water mixed with the ABTS^•+^ working solution was used as blank. Absorbance was determined at 734 nm using a UV-5100 spectrophotometer.

#### 2.5.2. Antihypertensive Activity

The ACE-inhibitory activity was estimated according to a previous method [26]. Peptides, N-[3-(2-Furyl)acryloyl]-Phe-Gly-Gly (FAPGG), and ACE were dissolved in 80 mM HEPES buffer (containing 300 mM NaCl, pH 8.2). Peptides (40 μL, 1 mg/mL) from Control or Sample were seeded in a 96-well plate. FAPGG (50 μL, 1 mM) and ACE (10 μL, 100 U/L) were added subsequently. HEPES buffer was used as a blank instead of the peptide samples. The mixture in the 96-well plate was pre-incubated at 37 °C for 5 min. Afterwards, the absorbance at 340 nm was determined at 37 °C for 30 min.

### 2.6. Determination of Total Amino Acids and Free Amino Acids

Peptides from Sample at 0 h, 1 h, 4 h, 24 h, and Control at 24 h were selected for amino acid analysis. Briefly, all samples were cooled to ambient temperature after being hydrolyzed for 22 h at 110 °C using 6 M HCl. Afterwards, 50 μL of the solution was placed in an evaporating dish at 60 °C for 2 h. The dried samples were then dissolved by adding 0.1 M HCl. Phenyl isothiocyanate (PITC, 0.1 M) and triethylamine (0.1 M) were added to the dissolved samples for PITC derivatization. The amino acid composition was measured by HPLC-loading on an Agilent AdvanceBio AAA analytical column (4.6 mm × 250 mm, 2.7 μm, Santa Clara, CA, USA). A UV detector operating at 254 nm was used to quantify the amino acids. A 10.0 μL volume of the sample was injected, and the flow rate was 1.0 mL/min. Amino acid standards were diluted using 0.1 M HCl to derive standard-curve equations for each amino acid peak area.

An automated amino acid analyzer (LA8080, Hitachi, Japan) coupled with a sulfonic acid cationic-resin separation column was used to determine the free amino acid content. The C18 column was activated by adding 5 mL of water and 5 mL of methanol. After dissolving the samples in 0.02 M HCL, they were centrifuged for 5 min at 3500× *g*. Afterwards, the supernatant was obtained and purified using a 0.45 μm microfiltration membrane for the measurement of free amino acid content.

### 2.7. Identification of the Modified Peptides

Aliquots from Sample at 0 h, 4 h, and 24 h were selected for the HPLC-MS/MS analysis. These samples were desalted by Pierce C18 Spin Tips. After being redissolved in solvent A (0.1% formic acid in water), the peptides were examined using an Orbitrap Q-Exactive Plus connected to an EASY-nanoLC 1200 system (Thermo Fisher Scientific, Waltham, MA, USA). The peptide sample (1 μL) was loaded onto an analytical column (Acclaim PepMap C18, 75 μm × 25 cm) and separated using a 60 min gradient, beginning at 2% buffer B (80% ACN with 0.1% FA) and increasing gradually to 35% in 47 min, 100% in 1 min, and then remaining at that concentration for 12 min. The flow rate of the column was maintained at 300 nL/min at 40 °C. The mass spectrometer was operated in the data-dependent acquisition (DDA) mode, which automatically alternated between the MS and MS/MS modes. Full-scan mass spectrometry (*m*/*z* 200–1800) surveys were obtained in Orbitrap at a 70,000 resolution. The peptides were selected into the collision unit and fragmented by high-energy collisional dissociation (HCD) with a normalized collection energy of 28%. 

The raw data collected from the LC-MS/MS comparison with the tilapia skin collagen database was analyzed using the PEAKS Studio software (Version 10.6, Bioinformatics Solutions Inc., Waterloo, ON, Canada). The database was downloaded from Uniport. The false discovery rate (FDR) was 1%. In this study, the dynamic modifications of oxidation (mass shift of +15.99 Da), di-oxidation (+31.99 Da), carbonylation (+12.98 Da), α-aminoadipic semialdehyde (AAS, −1.03 Da), γ-Glutamic semialdehyde (GGS, −43.05 Da), MDA (+54.01 Da), and 4-hydroxyl-2-nonenal (HNE, +156.12 Da) were analyzed. 

### 2.8. Statistical Analysis

All results were obtained in triplicate and expressed as mean ± SD. One-way analysis of variance (ANOVA, SPSS 27.0) was used to determine the evident difference between the variables. The difference between variables was significant when *p* < 0.05.

## 3. Results and Discussion

### 3.1. MW Distribution of CPs

The MW distribution of the collagen hydrolysates and CPs is shown in Table 1. The percentage of low-molecular-weight peptides (less than 1000 Da) in collagen hydrolysates was 83.26%, and many of those peptides were less than 500 Da (58.22%). In this study, dialysis was used to remove free amino acids from the collagen hydrolysate as well as to desalt. CPs were obtained by dialysis of the collagen hydrolysate; a decrease in the percentage of small-molecular-weight (<500 Da) compounds was observed after dialysis (58.22% vs 45.47%), while the percentage of fractions at 500–1000 Da and higher than 1000 Da increased to 30.12% and 24.42%, respectively.

### 3.2. Oxidation Dynamics of Lipid and CPs

#### 3.2.1. Lipid Oxidation

**Figure 1:**
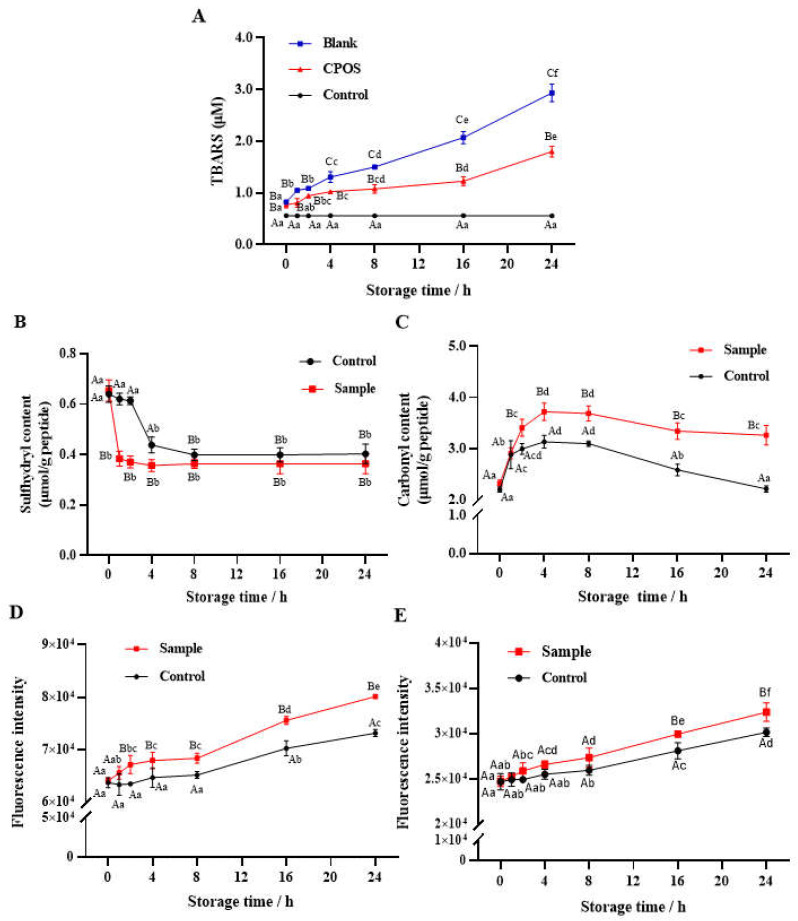
The TBARS value (**A**), sulfhydryl content (**B**), carbonyl content (**C**), Schiff base content (**D**) and dityrosine content (**E**) of Sample and Control during the 24 h storage at 37 °C. Blank: fish-oil emulsion; CPOS: fish-oil-fortified collagen-peptide emulsion; Control: collagen peptides in aqueous solution; Sample: the oxidized collagen peptides extracted from CPOS. Different capital letters indicate significant differences (*p* < 0.05) among different groups at the same time; different lowercase letters indicate significant differences (*p* < 0.05) at different storage times.

The results of lipid oxidation over 24 h in a fish-oil emulsion (Blank), a fish-oil-fortified collagen-peptide emulsion (CPOS), and an aqueous solution of collagen peptides (Control) stored at 37 °C are shown in Figure 1A. A trend of increasing TBARS values in Blank and CPOS was observed with the extension of storage time, while the TBARS values of Control did not change. Specifically, the amount of TBARS in Blank increased slowly within the initial 8 h, then rapidly (*p* < 0.05) increased during 8–24 h. However, TBARS in the lipid emulsion of CPOS was significantly (*p* < 0.05) lower than that of Blank due to the CP addition, a steady state observed during 4–16 h. Compared with Blank, TBARS in CPOS decreased by 22.1%, 28%, and 38.8% at 4 h, 8 h, and 24 h, respectively. 

During the process of lipid oxidation, a large amount of ROS is produced constantly [27], which can attack side-chain sites in amino acids and result in the oxidative damage of peptides. At the same time, many aldehyde compounds, such as MDA, are produced due to direct lipid oxidation [28]. These compounds are prone to binding to some amino acids, leading to a decrease in TBARS and oxidative damage of peptides. However, compared with Control, Blank and CPOS displayed a significant (*p* < 0.05) increase in the TBARS value, which suggested that this increase in TBARS levels in the emulsion was mainly caused by the rapid oxidation of fish oil.

#### 3.2.2. Sulfhydryl Content

The total sulfhydryl content of Sample and Control within 24 h is presented in Figure 1B. The sulfhydryl content of Sample decreased sharply (*p* < 0.05) within 1 h from 0.62 μmol/g peptide to 0.38 μmol/g peptide, and then no significant (*p* > 0.05) change was observed during 1 to 24 h. The sulfhydryl content of Control decreased only between 2–8 h from 0.61 μmol/g peptide to 0.40 μmol/g peptide. No significant (*p* > 0.05) difference was observed between Sample and Control from 8 to 24 h, indicating the depletion of sulfhydryl groups in the CPs. According to the result, lipid oxidation promoted a decrease in the sulfhydryl content of Sample compared with Control. Meanwhile, the sulfhydryl content of CPs in aqueous solution was consumed slowly, probably due to the formation of free radicals from the oxygen remaining in the headspace of the centrifuge tubes. The loss of sulfhydryl generally promoted the formation of disulfide bonds, which resulted in the cross-linking and aggregation of proteins [17].

#### 3.2.3. Carbonyl Content

The carbonyl content of Sample and Control within 24 h is shown in Figure 1C. The concentration–time curve of the carbonyl content in Sample and Control showed the same trend of increase, stability, and decline at 0–4 h, 4–8 h, and 8–24 h, respectively. Significant (*p* < 0.05) differences in carbonyl content were initially observed at 2 h, which then increased by 18.8%, 19.0%, and 47.5% at 4, 8, and 24 h, respectively.

According to Estévez et al. [28], one of the most notable indicators of oxidative damage to proteins is carbonylation. The induction of H_2_O_2_ is an essential step in carbonyl production [29]. There are two categories of carbonyls formed by oxidation: primary and secondary production (small-molecule aldehyde adductions) [30]. The on-site formation of primary carbonylation in proteins is usually due to ROS attack on the ε-amino groups of susceptible amino acids (Lys, Arg, and Pro) [28]. The small-molecule aldehydes (such as MDA) formed by lipid oxidation also show a pro-oxidation effect on peptides and form stable products by adduction reactions [28]. Therefore, that the significant decrease in carbonyl content (*p* < 0.05) observed in Sample at 8–16 h may be attributed to the further degradation of oxidation products. 

#### 3.2.4. Schiff Base

The fluorescence intensity of Schiff bases in Sample and Control within 24 h is shown in Figure 1D. The concentration–time curve of the Schiff base content in Control showed two periods of stability and increase at 0–8 h and 8–24 h, respectively, while that in Sample showed three periods of slow increase, steady, and rapid increase at 0–4 h, 4–8 h and 8–24 h, respectively. Compared with Control, a significant (*p* < 0.05) increase in the Schiff base content was observed in Sample after 2 h, which suggested that the oxidation reaction of CPs was significantly accelerated by fish oil and H_2_O_2_ in the emulsion. Furthermore, the increase in Schiff base content from 8 to 24 h was opposite to the decrease in carbonyls in Control and Sample, indicating that the peptides underwent further oxidative deterioration from carbonyls to Schiff bases. Schiff bases were previously reported to be one of the products of the further oxidation of carbonyl and are mainly produced in the late oxidation period [29]. The MDA can react with an ε-amino group in Lys to generate an unstable Schiff base, and another aldehyde group of the MDA may continue to react with Lys acids on other peptide chains, leading to cross-linking between peptide chains [31].

#### 3.2.5. Dityrosine

As shown in Figure 1E, the fluorescence intensity of dityrosine in Sample and Control increased with the extension of storage time. The content of the two groups accumulated slowly during 0–8 h, and increased rapidly during 8–24 h, indicating that the formation of dityrosine occurred mainly in the later period of oxidation. The content of dityrosine in both Sample and Control increased significantly (*p* < 0.05) during 16–24 h, and the dityrosine content of Sample was significantly (*p* < 0.05) higher than that of Control after storage for 8 h.

Dityrosine is an indicator of characteristic physicochemical alterations in proteins and is produced by the oxidation of Tyr through the formation of a tyrosyl phenoxyl radical [24]. The production of dityrosine was observed when myofibrillar proteins were subjected to Fenton’s reagent, which caused a decrease in proteolytic susceptibility through intermolecular cross-linking [32]. The dityrosine formation suggested that Tyr could be oxidized during storage and result in the formation of polypeptide polymers, which might lead to a negative impact on biological activities.

### 3.3. Changes in Biological Activity

#### 3.3.1. Antioxidant Activity

**Figure 2:**
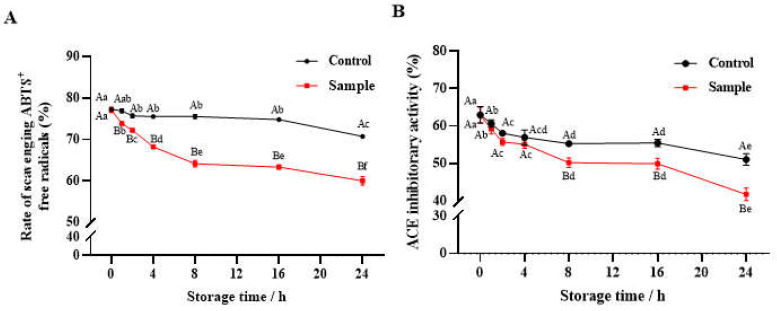
The ABTS·+ radical-scavenging capacity (**A**) and ACE-inhibitory activity (**B**) of Sample and Control during the 24 h storage at 37 °C. Control: collagen peptides in aqueous solution; Sample, the oxidized collagen peptides extracted from a fish-oil-fortified collagen-peptide emulsion. Different capital letters indicate significant differences (*p* < 0.05) among different groups at the same time; different lowercase letters indicate significant differences (*p* < 0.05) at different storage times.

The ABTS^•+^ radical-scavenging capacity of Control decreased slowly within 24 h, while the antioxidant activity of Sample decreased rapidly (*p* < 0.05) at 0–8 h and then decreased slowly at 8–24 h (Figure 2A). Compared with Control, the free-radical scavenging activity of Sample was significantly (*p* < 0.05) reduced by 9.1%, 15.1%, and 15.2% at 4, 8, and 24 h, respectively. Higher antioxidant activity was related to some hydrophilic amino acids or basic amino acids such as Tyr, His, Lys, and Arg, which could be an acceptor of the free radicals or react with superoxide anions [33]. Therefore, the oxidative modifications of these amino acids might result in the decline in antioxidant activity in the peptides.

#### 3.3.2. Antihypertensive Activity

The ACE-inhibitory activity of Sample and Control are shown in Figure 2B. Two periods of rapid and slow decline were observed in the ACE-inhibitory activity curve: 0–2 h and 2–24 h for the Control and 0–8 h and 8–24 h for the Sample, respectively. The presence of hydrophobic amino acid residues (Trp, Tyr, Phe, and Pro) at the C-terminus or N-terminus of the peptides could improve their ACE-inhibitory activity [34]. The decline of ACE-inhibitory activity in this study might be due to the oxidative modifications of these hydrophobic amino acids (HAAs).

### 3.4. Correlation Analysis between Oxidative Behavior and Biological Activity

The relationship between biological activity and oxidative behavior in collagen peptides has been seldom reported. To gain an insight into the relevance of the bioactivities of CPs toward oxidative indicators, a Pearson correlation analysis of the oxidative indicators and bioactivities of the peptides was performed at storage times of 0–4 h, 4–8 h, 8–24 h, and 0–24 h, respectively (Table 2).

The oxidation of peptides was divided into three stages according to the dynamic curves in the accelerated storage trail, which were early stage (0–4 h), middle (4–8 h), and later stage (8–24 h). At the early stage of oxidation, there was a significant (*p* < 0.01) correlation between each bioactivity and oxidative indicator, and the carbonyl content exhibited the strongest correlation with the antioxidant activity and ACE-inhibitory activity (r = −0.947, −0.911). The antioxidant and ACE-inhibitory activities were positively correlated with sulfhydryl content, while negative correlations with carbonyl content, Schiff base content, and dityrosine content were observed. 

At the middle stage of 4–8 h, no evident (*p* > 0.05) correlations were found between oxidation and bioactivity variables, and the antioxidant and ACE-inhibitory activities of Sample were still decreasing significantly (*p* < 0.05), while the oxidation indexes of Sample remained stable. It is noteworthy that the mid-stage of 4–8 h was the turning point of the oxidation-index curve, such as from an increase to a decrease in carbonyl content, or from a slow accumulation to a rapid increase in Schiff base content and dityrosine content. However, the increase in Schiff base and dityrosine content contributed to the decrease in bioactivities at the later stage of oxidation (8–24 h), because of a significant (*p* < 0.05 or *p* < 0.01) negative correlation between their contents and bioactivities.

According to Milic et al. [35], protein nucleophiles are altered by the highly reactive carbonyls (oxoLPPs) derived from lipid peroxidation by Schiff base production or Michael addition. Depending on the formation mechanism, peptide–oxoLPP adducts can carry aldehyde or keto groups and thus become part of the total carbonylation of proteins. Thus, a rapid increase in the carbonyls of CPs was observed at the early stage of oxidation (0–4 h) in this study. The conventional carbonyl-labeling methods will not detect the peptide-oxoLPP adducts if a carbonyl group has been removed during consecutive processes. Therefore, the carbonyl content of CPs decreased slowly at the later stage of oxidation (8–24 h). At the middle stage of oxidation (4–8 h), more CPs were modified by oxoLPPs through Michael addition, which contributed to the accumulation of carbonyls, while Michael adducts with carbonyl groups were further stabilized via cyclic hemiacetals. Hence, the generation and consumption of carbonyls reached an equilibrium, resulting in a steady state, although more and more CPs were oxidized during this period. 

During the whole storage time (0–24 h), each oxidative indicator showed a significant (*p* < 0.01) correlation with the bioactivities, indicating that oxidative behaviors lead to a decrease in the bioactivities of CPs, and this decrease coincided with the time at which different oxidation reactions occurred (a time-dependent change). At the early stage of oxidation, the carbonyl content is suitable as an oxidative indicator that is associated with the bioactivities of CPs due to the stronger correlation between the antioxidant activity and ACE-inhibitory activity (Table 2). However, at the later stage or whole oxidation process, the content of Schiff base or dityrosine is suitable as an oxidative indicator that is associated with the antioxidant or ACE-inhibitory activity of CPs.

Among the oxidation indicators (sulfhydryl, carbonyl, Schiff base, and dityrosine), the content of Schiff bases and dityrosine exhibited the strongest correlation (r > 0.84) during the whole storage time, except for the middle stage (4–8 h). In particular, significant correlations (*p* < 0.05 or *p* < 0.01) were observed among these oxidation indicators at the early stage (0–4 h). However, at the later stage of oxidation (8–24 h), only a significant (*p* < 0.05) correlation was observed between carbonyl and dityrosine (or Schiff base) content, as well as between the Schiff base and dityrosine content. 

Furthermore, there was a significant (*p* < 0.01) positive correlation between the antioxidant activity and the ACE-inhibitory activity (r = 0.943) during the whole storage time (0–24 h), suggesting that the dynamics of the loss of the two activities are consistent. 

### 3.5. Changes in Total Amino Acids and Free Amino Acids

Protein oxidation is associated with nutrition loss, which is expressed by a decline in amino acid content and an increase in oxidative modification [15]. Compared with proteins, the amino acids in the CPs were exposed and more likely to be attacked by free radicals. Five samples (0, 1, 4, and 24 h of Sample, and 24 h of Control, named Con-24 h) with different oxidation levels were selected to analyze the composition content of total amino acids and the free amino acids (Table 3).

Glycine (Gly, 162.96 mg/g), proline (Pro, 138.28 mg/g), hydroxyproline (Hyp, 80.53 mg/g), and glutamic acid (Glu, 67.62 mg/g) were abundant in CPs, which was consistent with the result of the previous study [1]. Compared with Sample at 0 h, the content of total amino acids in Con-24 h decreased significantly (*p* < 0.05), which suggested that the collagen peptides in aqueous solution also suffered modification of their amino acids due to oxidation. A significant (*p* < 0.05) decrease in total amino acids was also observed in Sample at 24 h compared with Con-24. Moreover, a dynamic decrease in amino acids was observed in Sample during storage. Compared with Sample at 0 h, a significant (*p* < 0.05) decrease in the content of Hyp, Arg, Met, and Lys was observed in Sample at 1 h, which indicated that they were the amino acids sensitive to being attacked by free radicals. His, Pro, and Tyr were significantly decreased, as well as Met and Lys, in Sample at 4 h. In Sample at 24 h, a continuous decline in the content of Hyp, His, Pro, and Met was observed. Overall, the content of HAAs decreased to a greater extent compared with the other amino acids.

The loss of Pro and Hyp could be attributed to attack by ROS and the addition of oxygen atoms to the side chain [28]. Arg and Lys are prone to undergoing deamination and carboxylation to form GGS and AAS [29], which is consistent with the increase in carbonyls. Moreover, the oxidation of Arg and Lys usually induces side-chain modification and can be identified by HPLC-MS/MS analysis [36]. Depletion of Met may occur because of mono-oxidation or higher levels of oxidative modifications [17]. Furthermore, the decrease in Tyr was consistent with the increase in dityrosine (Figure 1E), and the generation of dityrosine is a major reason for protein aggregation [37]. In addition, compared with Sample at 0 h, the content of basic amino acids (BAAs), aromatic amino acids (ARAAs), and HAAs decreased significantly (*p* < 0.05) in Sample at 24 h, indicating that these kinds of amino acids can also be attacked by free radicals and cause oxidative modification.

A previous study showed that peptides with low molecular weight and containing specific amino acid sequences (especially Lys-, -Gly-Phe-, and -Arg) had higher antioxidant activity [33]. Soy peptide fractions with high His, Tyr, and phenylalanine (Phe) content exhibited higher ABTS^•+^-radical scavenging activity [31]. The hypotensive activity of peptides is related to some HAAs [34]. Small peptides with the presence of HAAs, arginine, or valine at the C-terminus showed higher ACE-inhibitory potential [38]. According to this, it could be inferred that oxidation modifications of these amino acids could lead to a decrease in antioxidant or ACE-inhibitory activity, which is consistent with the results of this study.

The content of free amino acids in Control and Sample displayed a significant (*p* < 0.05) increase with the extension of storage time. Compared to Con-24 h (7.92 mg/g), the free amino acid content in Sample at 24 h (12.84 mg/g) increased by 62%. The result suggested that peptide bonds may be broken due to free-radical attack or that some peptides may be further degraded during oxidation, resulting in the generation of smaller peptides or free amino acids [36].

### 3.6. Identification of Modified Peptides

Proteomics was employed to further validate the oxidative modification of the collagen peptides. The peptides with high confidence intervals (≥85%) and high abundance (≥10^6^) in the database were screened, and the final database consisted of 2004 identified peptides in Sample at 0 h, 888 in Sample at 4 h, and 1080 in Sample at 24 h, respectively. In this database, the length of the collagen peptides varied from 4 to 25 amino acids, and they matched sequences from different tilapia skin proteins, including collagen type I alpha1, collagen type I alpha 1b, fibrillar collagen NC1-domain-containing protein, and other proteins. The proportions of modified and oxidized amino acid residues in the database were statistically analyzed, as shown in Table 4.

Eight oxidation modification types were observed in Sample, including oxidation, di-oxidation, GGS, AAS, AAA, MDA, carbonylation, and HNE. Notably, oxidative modification of amino acids was found in the Sample initially (0 h), which suggested that oxidative damage occurred during the preparation of CPs. Furthermore, oxidation and di-oxidation involved in some amino acids (Met, Phe, Arg, etc.) attained higher proportions compared with the other modifications, the sum of which was 20.46% (Sample at 0 h), 24.66% (Sample at 4 h), and 21.02% (Sample at 24 h), respectively. Compared with Sample at 0 h, the total modification proportions of GGS, AAS, AAA, MDA, carbonylation, and HNE increased by 72% (5.33% vs 9.18%) in the identified peptide database of Sample at 4 h. These modifications are closely related to the oxidation of basic amino acids (Lys and Arg) and the production of primary carbonyls, which is consistent with the decrease in Lys and Arg and the rapid increase in carbonyls of Sample at 4 h (Figure 1C). However, compared with Sample at 4 h, the percentage of these modification types declined in Sample at 24 h, which is consistent with the significant (*p* < 0.05) decrease in carbonyl content at the later stage.

The proportions of oxidized amino acid residues in the database were also analyzed. Met was the amino acid most sensitive to oxidation, followed by Lys and Arg. The proportion of oxidative Met reached up to 22.89% in Sample at 4 h. However, it dropped to 14.16% in Sample at 24 h, which may be due to the cross-linking of peptides leading to the generation of disulfide bonds and loss of sulfur-containing amino acids [39]. In addition, the proportion of other oxidative amino acids, such as Arg, Phe, and Asn, decreased in Sample at 24 h, likely due to aggregation of the peptides. The proportion of oxidative Lys and Arg reached up to 21.84% and 17.68% in Sample at 4 h, which was also as high as 18.8%, and 12.91%, respectively, in Sample at 0 h. Among the identified peptides, a high percentage of oxidative modifications was found in Phe, Pro, Asn, and Ala, most of which were involved in providing the peptide bioactivity [40]. Notably, the Hyp residue in CPs has the same mass as the oxidation products of Pro, and the mono-oxidation product of Pro was tagged as a Hyp in this study. The minor increase in the percentage of Hyp to Pro (O:P) suggested that a portion of Pro mono-oxidation was mistaken for Hyp, resulting in the mono-oxidation modifications of Pro being concealed.

Table 4 displays the oxidation sites of several modified peptides with the same amino acid sequence fragment in Sample at 0 h, 4 h, and 24 h. The oxidation sites were mainly observed at Arg (oxidation, di-oxidation, GGS formation, carbonylation, and MDA adducts), Lys (oxidation, di-oxidation, AAS and AAA formation, MDA and HNE adducts), and Met (oxidation, di-oxidation), which was consistent with the high proportion of oxidative amino acids. The changes in modified peptides provided specific information on the oxidative products in CPs induced by fish oil and H_2_O_2_.

## 4. Conclusions

The co-induced oxidation by fish oil and H_2_O_2_ accelerated the oxidative process of CPs, and the activities of antioxidation and ACE inhibition also decreased gradually due to oxidation deterioration during storage. The concentration–time curve of the carbonyls, Schiff base, and dityrosine in Sample and Control showed the same trends, with evident (*p* < 0.05) differences at the same storage time. At the early stage (0–4 h), Met, Lys, and Arg were oxidized by various types of oxidative modifications, including oxidation, di-oxidation, GGS, AAS, AAA, and HNE. Afterwards, a period of stability of oxidation indexes appeared, although the oxidation continued, and the bioactivities decreased. Then, the carbonyls were further oxidized and gradually consumed. Meanwhile, Schiff bases and dityrosine accumulated gradually and increased rapidly at 8–24 h, resulting in cross-linking between peptide chains. A high correlation between oxidative modification and the biological activity of collagen peptides was found (*p* < 0.01), for which the carbonyls could be a marker of primary oxidation, while Schiff bases and dityrosine played a major role in further oxidation at the later stage. This study has elucidated the underlying mechanism of collagen peptide oxidation that leads to a decrease in biological activity during storage.

## Figures and Tables

**Table 1:** MW distribution of collagen hydrolysates and CPs.

Component	Relative Content (%)		
	>1000 Da	500–1000 Da	<500 Da
Collagen hydrolysates	16.75	25.04	58.22
CPs	24.42	30.12	45.47

NOTE: CPs, collagen peptides.

**Table 2:** Pearson correlation coefficient for the oxidative behaviors and bioactivities of Sample.

Time	Variables	Sulfhydryl	Carbonyl	Schiff Base	Dityrosine	Antioxidant	Antihypertensive
**0–4 h**	Sulfhydryl	\	−0.830 **	−0.646 *	−0.677 *	0.762 **	0.832 **
	Carbonyl		\	0.721 **	0.739 **	−0.947 **	−0.911 **
	Schiff base			\	0.842 **	−0.754 **	−0.717 **
	Dityrosine				\	−0.777 **	−0.800 **
	Antioxidant					\	0.858 **
	Antihypertensive						\
**4–8 h**	Sulfhydryl	\	0.674	0.472	−0.036	0.134	0.028
	Carbonyl		\	−0.217	−0.480	0.304	0.150
	Schiff base			\	0.279	−0.036	0.04
	Dityrosine				\	−0.549	−0.630
	Antioxidant					\	0.964 **
	Antihypertensive						\
**8–24 h**	Sulfhydryl	\	0.104	0.096	0.059	0.242	−0.107
	Carbonyl		\	−0.707 *	−0.790 *	0.542	0.618
	Schiff base			\	0.939 **	−0.820 **	−0.779 *
	Dityrosine				\	−0.801 **	−0.865 **
	Antioxidant					\	0.860 **
	Antihypertensive						\
**0–24 h**	Sulfhydryl	\	−0.767 **	−0.405	−0.422	0.613 **	0.565 **
	Carbonyl		\	0.302	0.303	−0.623 **	−0.516 *
	Schiff base			\	0.969 **	−0.863 **	−0.895 **
	Dityrosine				\	−0.882 **	−0.933 **
	Antioxidant					\	0.943 **
	Antihypertensive						\

NOTE: Sample, the oxidized collagen peptides extracted from a fish-oil-fortified collagen-peptide emulsion during the 24 h storage at 37 °C. *, correlation with *p* < 0.05; **, correlation with *p* < 0.01.

**Table 3:** The amino acid composition of collagen peptides in Sample or Control stored at 37 °C for different times (results are expressed as mg/g peptides).

Type ^C^	Amino Acid ^B^	Sample ^A^				Control ^A^
		0 h	1 h	4 h	24 h	Con-24 h
**Total**	Asp	43.24 ± 1.45 ^ab^	45.20 ± 0.63 ^a^	42.79 ± 0.23 ^ab^	43.50 ± 0.44 ^ab^	42.24 ± 0.36 ^b^
	Glu	67.62 ± 0.67 ^b^	71.04 ± 0.07 ^a^	67.09 ± 0.59 ^b^	70.63 ± 0.16 ^a^	66.85 ± 0.15 ^b^
	Hyp	80.53 ± 0.16 ^a^	78.20 ± 0.65 ^b^	78.03 ± 0.49 ^b^	73.41 ± 0.65 ^c^	77.49 ± 0.05 ^b^
	Cys-Cys	0.28 ± 0.01	0.30 ± 0.03	0.31 ± 0.06	0.27 ± 0.03	0.27 ± 0.07
	Ser	22.63 ± 0.02 ^c^	24.75 ± 0.13 ^a^	23.61 ± 0.16 ^b^	24.69 ± 0.07 ^a^	22.76 ± 0.58 ^bc^
	Gly	162.96 ± 0.77 ^bc^	168.58 ± 0.82 ^a^	161.56 ± 1.87 ^c^	167.24 ± 0.15 ^ab^	161.16 ± 1.66 ^c^
	His	5.50 ± 0.09 ^ab^	5.69 ± 0.07 ^a^	5.47 ± 0.00 ^b^	5.09 ± 0.05 ^c^	5.53 ± 0.04 ^ab^
	Arg	58.96 ± 0.27 ^a^	57.12 ± 0.60 ^bc^	55.82 ± 0.57 ^c^	56.03 ± 0.51 ^bc^	57.57 ± 0.26 ^ab^
	Thr	15.84 ± 0.53 ^a^	14.56 ± 0.35 ^ab^	14.01 ± 0.25 ^b^	14.16 ± 0.01 ^ab^	14.44 ± 0.80 ^ab^
	Ala	59.39 ± 0.39 ^bc^	61.25 ± 0.18 ^a^	58.34 ± 0.60 ^c^	60.39 ± 0.25 ^ab^	58.78 ± 0.79 ^bc^
	Pro	138.28 ± 2.73 ^a^	133.62 ± 1.20 ^a^	121.93 ± 0.03 ^b^	93.86 ± 0.98 ^c^	136.45 ± 0.64 ^a^
	Tyr	2.40 ± 0.14 ^a^	2.25 ± 0.02 ^a^	0.98 ± 0.06 ^bc^	0.88 ± 0.02 ^c^	1.15 ± 0.01 ^b^
	Val	11.41 ± 0.09 ^ab^	11.82 ± 0.26 ^a^	10.96 ± 0.01 ^b^	11.42 ± 0.63 ^ab^	11.09 ± 0.10 ^b^
	Met	4.12 ± 0.05 ^b^	3.65 ± 0.09 ^c^	3.12 ± 0.11 ^e^	3.37 ± 0.04 ^d^	5.31 ± 0.05 ^a^
	Ile	5.46 ± 0.16	5.59 ± 0.07	5.39 ± 0.06	5.41 ± 0.08	5.40 ± 0.04
	Leu	12.47 ± 0.41	12.98 ± 0.39	12.02 ± 0.22	12.55 ± 0.13	12.42 ± 0.37
	Phe	11.67 ± 0.29	11.88 ± 0.09	11.41 ± 0.17	11.74 ± 0.16	11.39 ± 0.04
	Lys	28.88 ± 0.02 ^a^	27.77 ± 0.54 ^b^	25.91 ± 0.49 ^c^	26.11 ± 0.23 ^bc^	27.60 ± 0.61 ^ab^
	BAAs	93.33 ± 0.38 ^a^	90.58 ± 1.21 ^a^	87.21 ± 1.05 ^b^	87.23 ± 0.79 ^b^	90.71 ± 0.90 ^a^
	AAAs	110.86 ± 2.12 ^bc^	116.24 ± 0.70 ^a^	109.89 ± 0.82 ^c^	114.12 ± 0.28 ^ab^	109.09 ± 0.20 ^c^
	ARAAs	14.07 ± 0.43 ^a^	14.13 ± 0.12 ^a^	12.39 ± 0.11 ^b^	12.62 ± 0.18 ^b^	12.54 ± 0.05 ^b^
	HAAs	242.18 ± 1.51 ^a^	240.78 ± 1.78 ^a^	223.16 ± 1.09 ^b^	198.75 ± 0.86 ^c^	240.84 ± 1.73 ^a^
	Total	731.63 ± 1.86 ^a^	736.24 ± 3.22 ^a^	698.75 ± 4.14 ^c^	680.76 ± 0.10 ^d^	717.87 ± 5.88 ^b^
**Free**	Total	3.62 ± 0.03 ^e^	6.81 ± 0.05 ^d^	7.52 ± 0.04 ^c^	12.84 ± 0.04 ^a^	7.92 ± 0.01 ^b^

NOTE: ^A^, the 0 h, 1 h, 4 h, and 24 h samples are the oxidized collagen peptides extracted from a fish-oil-fortified collagen-peptide emulsion stored at 37 °C for 0 h, 1 h, 4 h, and 24 h. The sample of Con-24 h is of collagen peptides stored at 37 °C for 24 h. ^B^, BAAs: basic amino acids, including Arg, His, and Lys; AAAs: acidic amino acids, including Asp and Glu; ARAAs: aromatic amino acids, including Tyr, Trp, and Phe; HAAs: total hydrophobic amino acids, including Ala, Pro, Trp, Val, Met, Ile, Leu, and Phe. ^C^, Total: hydrolyzed amino acid composition; Free: free amino acid content. Different lowercase letters indicate significant (*p* < 0.05) differences in the same row.

**Table 4:** The proportion of oxidative modifications, oxidized amino acid residues, and common peptide segments in the identified peptide sequence database for Sample stored at different times (%).

Sample ^a^		0 h	4 h	24 h
**Modifications ^b^**	Oxidation	9.78	14.30	12.41
	Di-oxidation	10.68	10.36	8.61
	GGS	2.59	4.84	3.61
	AAS	1.35	1.46	1.11
	AAA	0.75	0.90	0.46
	MDA	0.34	0.85	0.65
	Carbonylation	0.15	0.68	0.19
	HNE	0.15	0.45	0.19
**Sensitive amino acid residue**	Met (M)	7.17	22.89	14.16
	Lys (K)	18.80	21.84	22.48
	Arg (R)	12.91	17.68	14.81
	Phe (F)	2.98	6.59	4.55
	Pro (P)	2.44	2.76	2.27
	Asn (N)	1.61	1.95	1.72
	Ala (A)	0	0.15	0.07
	O:P	40.17	41.71	41.36
**Common peptide segment ^c^**	PGPGP**M**	PGPGPM	PGPGPM(+15.99)	PGPGPM(+15.99)
	PGA**R**	PGAR(+15.99)-X	PGAR(+15.99)-X	PGAR(+54.01)-X
	GPAGP**R**	GPAGPR(+31.99)-X	GPAGPR(−43.05)-X	GPAGPR(−43.05)-X
	**K**APDPFR	KAPDPFR	K(−1.03)APDPFR	K(+14.96)APDPFR
	**K**APDPLR	K(−1.03)APDPLR-X	K(+14.96)APDPLR-X	K(+14.96)APDPLR-X
	GPTG**R**	X_1_- GPTGR-X_2_	X_1_- GPTGR(+12.98)-X_2_	X_1_- GPTGR(−43.05)-X_2_
	**K**GPSG	X_1_- KGPSG-X_2_	X_1_- KGPSG-X_2_	X_1_-K(+156.12) GPSG -X_2_

NOTE: (a) The 0 h, 4 h, and 24 h samples refer to the oxidized collagen peptides extracted from a fish-oil-fortified collagen-peptide emulsion stored at 37 °C for 0 h, 4 h, and 24 h. The proportion of modifications is the number of peptides of each modification to the total number of peptides. The proportion of sensitive amino acid oxidation is the number of each oxidized amino acid residue to the total number of that amino acid residue. (b) GGS: γ-glutamic semialdehydes, AAS: α-aminoadipic semialdehydes, AAA: α-aminoadipic acid, MDA: malonaldehyde, HNE: 4-hydroxy-2-nonenal. (c) Amino acid with an underline indicates a modified amino acid; X, X_1_, and X_2_ represent different amino acid residues at the N- or C-terminus of the peptides.

## Data Availability

The datasets generated for this study are available on request to the corresponding author.
